# Rapid Microbiological Testing: Monitoring the Development of Bacterial Stress

**DOI:** 10.1371/journal.pone.0013374

**Published:** 2010-10-14

**Authors:** Boris Zavizion, Zhihui Zhao, Aphakorn Nittayajarn, Ronald J. Rieder

**Affiliations:** BioSense Technologies, Inc., Woburn, Massachusetts, United States of America; Texas A&M, United States of America

## Abstract

The ability to respond to adverse environments effectively along with the ability to reproduce are *sine qua non* conditions for all sustainable cellular forms of life. Given the availability of an appropriate sensing modality, the ubiquity and immediacy of the stress response could form the basis for a new approach for rapid biological testing. We have found that measuring the dielectric permittivity of a cellular suspension, an easily measurable electronic property, is an effective way to monitor the response of bacterial cells to adverse conditions continuously. The dielectric permittivity of susceptible and resistant strains of *Escherichia coli* and *Staphylococcus aureus*, treated with gentamicin and vancomycin, were measured directly using differential impedance sensing methods and expressed as the Normalized Impedance Response (NIR). These same strains were also heat-shocked and chemically stressed with Triton X-100 or H_2_O_2_. The NIR profiles obtained for antibiotic-treated susceptible organisms showed a strong and continuous decrease in value. In addition, the intensity of the NIR value decrease for susceptible cells varied in proportion to the amount of antibiotic added. Qualitatively similar profiles were found for the chemically treated and heat-shocked bacteria. In contrast, antibiotic-resistant cells showed no change in the NIR values in the presence of the drug to which it is resistant. The data presented here show that changes in the dielectric permittivity of a cell suspension are directly correlated with the development of a stress response as well as bacterial recovery from stressful conditions. The availability of a practical sensing modality capable of monitoring changes in the dielectric properties of stressed cells could have wide applications in areas ranging from the detection of bacterial infections in clinical specimens to antibiotic susceptibility testing and drug discovery.

## Introduction

It has been well documented that the initiation of the stress response in a bacterial cell occurs within minutes after exposure to both non-specific (heat, acids, peroxides) and specific (antibiotics) stress conditions [1], [2], [3], [4]. Given the availability of an effective and simple sensing modality, the immediacy and ubiquity of this response could form an attractive foundation for the development of a new approach to microbiological testing.

In our previously studies, we hypothesized that monitoring the dielectric permittivity of a cell suspension is a simple, non-invasive, and practical method for sensing the physiological changes which take place in stressed bacterial cells [5], [6]. The dielectric permittivity is an easily measurable electronic property obtained by recording the reactive component of the electrical impedance (i.e. capacitance) of the sample. The inclusion of differential impedance sensing methods in our system enables the signal from stressed bacteria to be discriminated and detected in complex samples.

The present study was undertaken to correlate changes in the dielectric properties of Gram-negative *Escherichia coli* (*E. coli*) and Gram-positive *Staphylococcus aureus* (*S. aureus*) exposed to different stress-generating conditions with the induced stress responses. Data are presented for susceptible/resistant strains of *E. coli* and *S. aureus* treated with gentamicin/ciprofloxacin and vancomycin/methicillin, respectively, to induce specific antibiotic stress responses. *E. coli* and *S. aureus* were also treated with Triton X-100 or H_2_O_2_ to induce chemical stress as well as being heat-treated to induce heat shock.

## Results

### MIC Results

Susceptibility testing revealed that the minimum inhibitory concentration (MIC) of gentamicin for *E. coli* (ATCC 700926) was 1.25 µg/ml. The corresponding MICs of ciprofloxacin and gentamicin were >64 µg/ml and 1 µg/ml, respectively, for the *E. coli* strain (M61965) having known resistance to ciprofloxacin.

The MIC of vancomycin for *S. aureus* (ATCC 29213) was 2 µg/ml in our hands and is consistent with the value (2 µg/ml) reported by Muthaiyan et al. [7]. The MICs of vancomycin and methicillin for MRSA (ATCC BAA-44) were 1.6 µg/ml and >600 µg/ml, respectively.

### Impedance characterization of culture media

**Figure 1:**
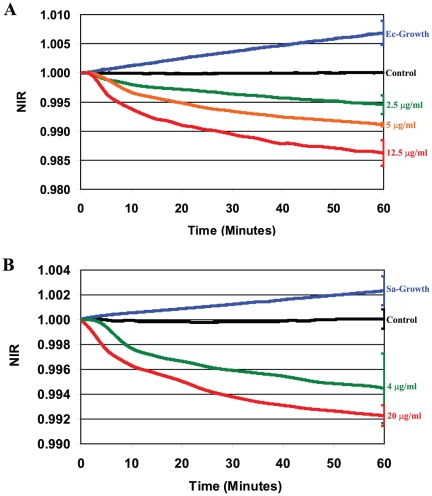
NIR profiles obtained for antibiotic-treated susceptible cells and for healthy growing cells. **A**) NIR profiles for *E. coli* were recorded during growth (Ec-Growth) and in the presence of 2.5 µg/ml, 5 µg/ml, and 12.5 µg/ml of gentamicin; **B**) NIR profiles for *S. aureus* were recorded during growth (Sa-Growth) and in the presence of 4 µg/ml and 20 µg/ml of vancomycin. Medium without cells (Control) was used as a negative control.

**Figure 2 pone-0013374-g002:**
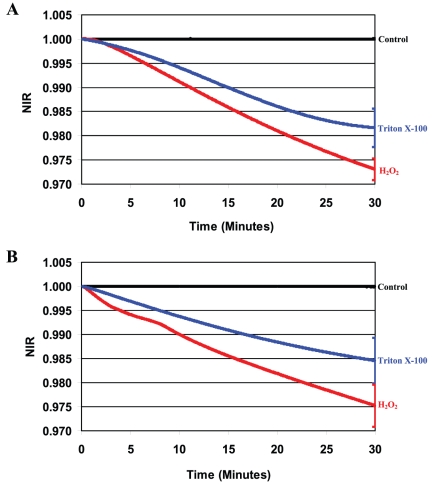
NIR profiles obtained for chemically stressed bacteria. Bacterial suspension was incubated in the presence of 0.5% Triton X-100 (blue curves) or 10 mM H_2_O_2_ (red curves). Medium without cells (Control) was used as a negative control (black curves). **A**) 8.2×10^5^ cfu/ml *E. coli* in LB **B**); 6.8×10^5^ cfu/ml *S. aureus* in TSB.

**Figure 3 pone-0013374-g003:**
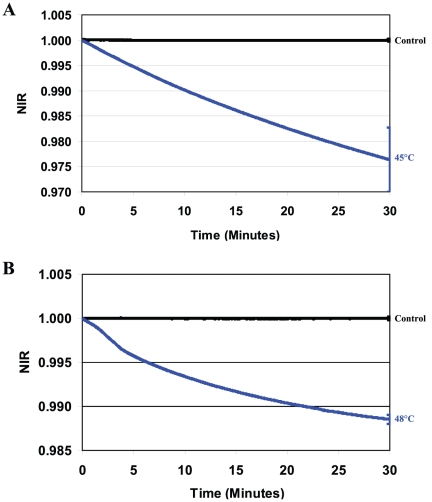
NIR profiles obtained for heat-shocked bacteria. **A**) *E. coli* (2.4×10^5^ cfu/ml) heat-shocked for 30 min at 45°C and re-suspended in LB; **B**) *S. aureus* (3.6×10^5^ cfu/ml) heat-shocked for 30 min at 48° and re-suspended in TSB. Media without cells (Control) were used as a negative control (black curves).

**Figure 4:**
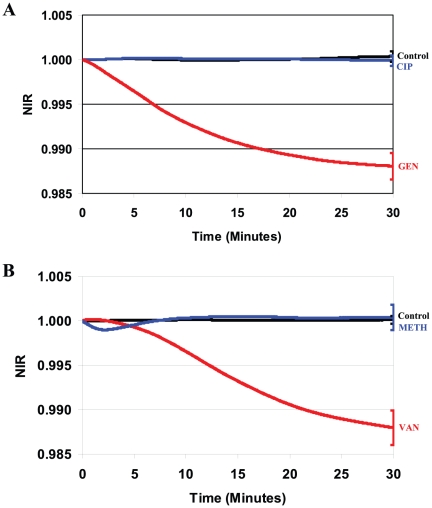
NIR profiles obtained for clinical isolates with antibiotic resistance. **A**) A clinical isolate of *E. coli* resistant to ciprofloxacin (CIP) was incubated in the presence of 45 µg/ml of CIP (blue curve) and 15 µg/ml of gentamicin to which it is susceptible (red curve). **B**) A clinical isolate of *S. aureus* (MRSA, Iberian isolate) was incubated in the presence of 400 µg/ml of methicillin (blue curve) and 8 µg/ml of vancomycin to which it is susceptible (red curve). Medium without cells (Control) was used as a negative control (black curve).

In a first series of experiments, both detection chambers were filled with the same medium and the NIR profiles for the different media used in this study were recorded to monitor the electrochemical behavior of sterile culture media with different supplements (Figures 1, 2, 3, and 4 - “Control”). As expected, in all cases, the NIR profiles of sterile media (with or without supplements) showed no significant variations from a constant value throughout the duration of the measurements.

### Impedance responses during bacterial growth

The NIR profiles of untreated growing bacterial cells are plotted in Fig 1. As illustrated in Figure 2A (curve “Ec-Growth”) and Figure 1B (curve “Sa-Growth”), progressive increases in the NIR values were recorded during the entire 60 min of incubation for both species. These results are consistent with the related impedance measurements reported by Colvin et al. and Wu et al. [8], [9] for *E. coli* and *S. aureus* cells, respectively, growing in broth.

Corresponding bacterial cell counts were obtained in parallel with the impedance measurements and the results are listed in Table 1. For both *E. coli* and *S. aureus*, overall increases in cell numbers were measured over the 60 min incubation period.

**Table 1:** The survival of *S. aureus* and *E. coli* treated with vancomycin and gentamicin.

Time (min)	*S. aureus* (Log_10_ CFU/ml)*				*E. coli* (Log_10_ CFU/ml)*			
	No Antibiotic		Vancomycin (20 µg/ml)		No Antibiotic		Gentamicin (5 µg/ml)	
	Mean	±SD	Mean	±SD	Mean	±SD	Mean	±SD
0	6.24	0.05	6.23	0.05	6.48	0.04	6.43	0.04
10	6.40	0.04	6.29	0.04	6.47	0.04	5.96	0.05
20	6.50	0.03	6.33	0.04	6.46	0.04	5.30	0.04
30	6.54	0.03	6.29	0.04	6.50	0.03	4.40	0.04
40	6.68	0.03	6.21	0.05	6.54	0.03	3.73	0.08
50	6.73	0.03	6.15	0.05	6.63	0.03	3.08	0.18
60	6.93	0.05	6.21	0.05	6.68	0.03	3.11	0.18

*Average of 3 experiments.

Interestingly, although no statistically significant changes in cell numbers were found for *E. coli* during the first 30 min of incubation, a detectable increase in NIR values was recorded.

### Impedance response from antibiotic-stressed susceptible bacteria

NIR profiles were obtained for *E. coli* and *S. aureus* during incubation with gentamicin and vancomycin, respectively, over a 60 min measurement period. For *E. coli*, individual profiles were obtained for three concentrations of gentamicin (2.5, 5 and 12.5 µg/ml) while *S. aureus* cells were treated with two concentrations of vancomycin (4 and 20 µg/ml). These results are plotted in Figure 1A and Figure 1B, respectively. For all five curves, the NIR profiles were qualitatively similar continuously decreasing in value irrespective of the nature of the species or drugs. These decreases are opposite in slope to the gradual increases observed for the healthy growing bacteria (curves “Ec-Growth” and “Sa-Growth”).

For both bacteria/antibiotic combinations, increasing the concentration of the antibiotic in the medium resulted in NIR profiles with increased negative slopes indicating a more intense response.

To ensure that the presence of the antibiotic *per se* did not bias the shape of the curves, NIR profiles of the respective media containing the highest concentration of each relevant antibiotic but no cells were measured. The NIR profiles obtained for all antibiotics were constant in value over time indicating no measurable electrochemical effects due to the antibiotic interacting with the medium or electrodes that could affect the shape of the NIR profiles involving cells.

The corresponding cell numbers for the antibiotic-treated suspensions were obtained in parallel with their NIR measurements and are again listed in Table 1. Overall, the plate counts showed a steady decrease in the number of colony forming units of *E. coli* in the presence of 5 µg/ml of gentamicin. However, these numbers reflect only those cells that were able to recover from injuries caused by the drug and form colonies on agar. In order to determine the number of cells viable at the time of measurement, acridine orange differential staining was conducted in parallel [10]. This revealed that more than 95 percent of *E. coli* cells were alive after 30 min of treatment decreasing to 88–90 percent by the end of incubation (60 min). In contrast, a 3-log reduction in population was detected by colony plate counts at 60 min.

A similar trend in cell viability was found for *S. aureus* treated with 20 µg/ml of vancomycin. Plate counts indicated little or no decrease in the number of colony forming units of *S. aureus* in the presence of the antibiotic. In parallel measurements, acridine orange staining confirmed that more than 97 percent of staphylococcal cells were alive after 30 min decreasing to nearly 80 percent at 60 min of incubation.

### Impedance responses during or after environmental stresses

In the next series of experiments, changes in the dielectric permittivities of cell suspensions subjected to chemical stresses and heat shock were studied. As seen in Figures 2 and 3, the NIR profiles for bacteria chemically stressed during constant exposure to Triton X-100 or hydrogen peroxide and for cells recovering from heat shock all decreased in value and are qualitatively similar to the profiles obtained for antibiotic-stressed cells. No changes in cell numbers after heating (45°C for *E. coli*, 48°C for *S. aureus*) or treatment with 0.5% Triton X-100 or 10 mM H_2_O_2_ were detected during the measurement period.

### Impedance response from antibiotic-resistant strains

Finally, experiments were conducted with ciprofloxacin-resistant *E. coli* and methicillin-resistant *S. aureus* (MRSA). In these experiments, the NIR value was calculated by directly comparing the dielectric permittivity of the antibiotic-treated cells with the dielectric permittivity of identical but untreated cell suspension. The corresponding NIR profiles reflect changes in the dielectric permittivities of the cell exclusively caused by exposure to the antibiotic. NIR profiles for medium with antibiotic (sterile medium only in reference chamber) and NIR profiles for untreated cells in both chambers served as controls for these experiments. As illustrated in Figures 4A and 4B, for the latter control, no significant deviations in NIR values were measured as expected when the same untreated cells were in both chambers. Similarly, when ciprofloxacin was added to the test chamber containing *E. coli* resistant cells or methicillin was added to MRSA cells, no changes in NIR values were recorded indicating that the corresponding dielectric permittivities of the antibiotic-treated resistant cell suspension were the same as for the non-treated resistant cells. In contrast, the addition of 15 µg/ml of gentamicin (*E. coli*) or 8 µg/ml of vancomycin (MRSA), to which these strains are susceptible, resulted in a negative sloping NIR profile qualitatively similar to the profiles found for all other susceptible cells.

### Statistical Analysis

An analysis was performed to determine the statistical significance of the differences in the NIR values obtained for the strains of *E. coli* and *S. aureus* exposed to the different stressors. Since the NIR profiles were measured continuously, average values of the NIR and the corresponding standard deviations (SD) were calculated at 10 minutes time intervals and are provided in Table S1. For clarity, error bars are plotted at the end points of each curve in Figs. 1–4 and are representative of the uncertainties for the entire curve.

The average NIR values for *E. coli* (ATCC 700926) treated with gentamicin and *S. aureus* (ATCC 29213) treated with vancomycin at a single time point (60 min) are listed in Table 2 along with the respective p-values using the *t*-test. For both strains, the difference between the mean NIR value for the control (medium with no cells) and the respective antibiotic treated cells was highly significant (p<0.002) irrespective of the concentration used. The statistical significance of the differences in NIR values obtained for the other strain/stressor combinations were also determined and were similar in value.

**Table 2:** The mean NIR values* for antibiotic-treated *E. coli* and *S. aureus.*

Antibiotic/Strain	Mean ± SD	P-value
***E. coli*** **/Gentamicin:**		
Control†	1.00000±0.00007	-
2.5 µg/ml	0.99443±0.00157	0.0018
5.0 µg/ml	0.99108±0.00029	<0.0001
12.5 µg/ml	0.98808±0.00163	0.0001
EC-Growth	1.00745±0.00060	<0.0001
***S. aureus*** **/Vancomycin:**		
Control†	1.00007±0.00079	-
4.0 µg/ml	0.99448±0.00280	0.0145
20.0 µg/ml	0.99255±0.00047	<0.0001
SA-Growth	1.00233±0.00118	0.0253

All measurements were conducted in triplicate (N = 3).

*Only for values recorded during the 60^th^ min of measurement.

† Medium without cells was used as a negative control.

## Discussion

In our experiments, decreases in the dielectric permittivity were routinely detected for *E. coli* and *S. aureus* immediately after the addition of an antibiotic to which they are susceptible with an intensity that is proportional to the antibiotic concentration used. In contrast, the dielectric permittivity increased for these same cells when no antibiotic was added.

The studies of Kohanski et al.[11] and Muthaiyan et al.[7] have revealed that treatment of these same strains of *E. coli* with gentamicin and *S. aureus* with vancomycin induced distinctive stress responses in these cells. Based on the results of transcriptional profiling, these stress responses were predominantly associated with oxidative stress (*E. coli*) and cell wall stress (*S. aureus*), respectively, and both were detected within 30 min or less after the onset of treatment. Comparison of our data with these results indicates that activation of the antibiotic-triggered stress responses is concomitant with instantaneous and continuous decreases in the dielectric permittivities of the cell suspension as reflected in the corresponding NIR values.

Furthermore, other investigators have shown that the activation of drug-induced stress response genes are dose-dependent [12], [13]. This finding is consistent with our data which show that increases in drug concentration result in NIR profiles having more pronounced intensity.

As part of our study, changes in the dielectric permittivities of clinical isolates of *E. coli* and *S. aureus* also susceptible to gentamicin and vancomycin but resistant to ciprofloxacin and methicillin, respectively, were measured. As seen in Figure 4A and 4B, the NIR profiles for the clinical isolates treated with gentamicin and vancomycin have negative slopes and are consistent with the profiles for the ATCC susceptible strains (Figure 1A and 1B). However, the NIR profiles obtained for these same isolates when treated with antibiotics to which they are resistant, are indistinguishable from the profiles found for unstressed bacteria while contrasting with the profiles for antibiotic-susceptible cells.

It has been shown previously that heat shock at 45°C (*E. coli*) or 48°C (*S. aureus*) as well as treatment with Triton X-100 or H_2_O_2_ activate stress responses in *E. coli* [14] and *S. aureus* [15] cells. Interestingly, the dielectric responses from heat shocked cells where the insult has already occurred (Figure 3A and 3B), have the same characteristics as those for the chemical- or antibiotic-stressed cells under constant pressure.

Taken collectively, all NIR profiles recorded for these two intentionally stressed bacteria were qualitatively similar, displaying decreasing values over the course of the measurements irrespective of the type of stress response, quantity of stressor, nature of stressor, or species. These characteristic NIR profiles were also observed in our earlier analysis of dielectric responses from heat-shocked at 50°C *E. coli* and Triton X-100 injured bacteria in contaminated platelets [5], responses from other bacterial species treated with antimicrobials having different mechanisms of action [6], as well as non-specific stress conditions including osmotic and hypoxic shock studied in our laboratory. Consequently, we conclude that the observed negative sloping NIR profiles are directly correlated with the development of the respective cellular stress responses.

### Non-invasive measurements of the development of stress

Historically, impedance sensing and its different components have been used as an electronic analog of the Petri dish to monitor the proliferation of organisms [8]. To the best of our knowledge, the present investigation is the first to demonstrate that the development of stress in susceptible bacteria in the presence of an antibiotic or other stressor can be monitored non-invasively by measuring the dielectric properties of a bacterial suspension.

Interestingly, no significant decreases in the numbers of viable cells were found during the first 30 min of exposure to the different stress conditions (as measured by colony count and/or acridine orange staining) suggesting that the changes in the value of the dielectric permittivity do not simply follow changes in the viable cell population.

The dielectric permittivity is a measure of the overall polarizability of both inorganic and organic molecules within the cell suspension. The use of differential sensing ensures that the resulting dielectric permittivities are restricted to cumulative changes associated with cellular processes only. The data presented in this report correlate cellular processes activated in response to stress with a decrease in dielectric permittivity and hence reduced polarizability. Furthermore, these dielectric changes are observed to be opposite in slope and occur on short timescales compared with changes associated with unsynchronized cell growth. Cellular activities potentially affecting the polarizability include a decrease in regular protein synthesis and an increase in the synthesis of stress response proteins, changes in the cell membrane potential and associated ion concentrations, inhibition of DNA replication, changes to DNA and protein conformational structures, and other processes associated with the conservation of energy and the restoration of damages in the cells quest to survive. The exact contribution to the dielectric properties of the multitude of intra- and extra-cellular biochemical interactions remains an open question.

In conclusion, the results presented here demonstrate that the bacterial response to stress causes specific changes to the dielectric permittivity of a suspension. These changes can be easily monitored using differential impedance sensing. Existing methods used to detect the development of stress are based on cell disruption and the molecular analysis of specific genes or proteins and require technically skilled personnel, extensive sample preparation including the use of radio isotopes and fluorescent labeling. The method reported here is simple, non-invasive, label-free, and provides results in real-time.

The approach is especially useful for determining drug susceptibility profiles rapidly enabling the prescription of targeted therapies. The ability to obtain results quickly has particular value for very slow growing pathogens such as mycobacteria [6]. The technique has now been applied to the detection of bacteria in complex biological specimens such as blood products [5]. In addition, vertically oriented electrodes, standard in many commercially available impedance systems, can be integrated into a format amenable to 96-well plates enabling application to the screening of large numbers of compounds as potential new drugs. Finally, the technique is readily extended to the rapid testing of cell types other than bacteria.

## Materials and Methods

### Media, antibiotics, organisms, culture conditions

All bacterial species were obtained from the American Type Culture Collection (ATCC, Manassas, VA) and Lawrence General Hospital (Lawrence, MA). *Staphylococcus aureus* (*S. aureus*) strains ATCC 29213 and methicillin-resistant ATCC BAA-44 (MRSA) were routinely maintained in Tryptic Soy Broth (TSB, Sigma, St. Louis, MO). *Escherichia coli* (*E. coli*) strains ATCC 700926 and ciprofloxacin resistant clinical isolate (M61965) were maintained in LB (ATCC, Manassas, VA). Vancomycin, gentamicin, methicillin, and ciprofloxacin were purchased from Sigma.

To heat shock the bacterial cells, mid-log phase cells were collected by centrifugation, re-suspended in PBS, and incubated for 30 min at 45°C (*E. coli*) or 48°C (*S. aureus*) as described [14], [15]. For the chemical stressor study, bacteria from mid-log phase were treated in their corresponding medium with stress-inducing concentrations of Triton X-100 (0.5%) or H_2_O_2_ (10 mM) as described [14], [16], [17].

### Impedance sensing hardware

**Figure 5:**
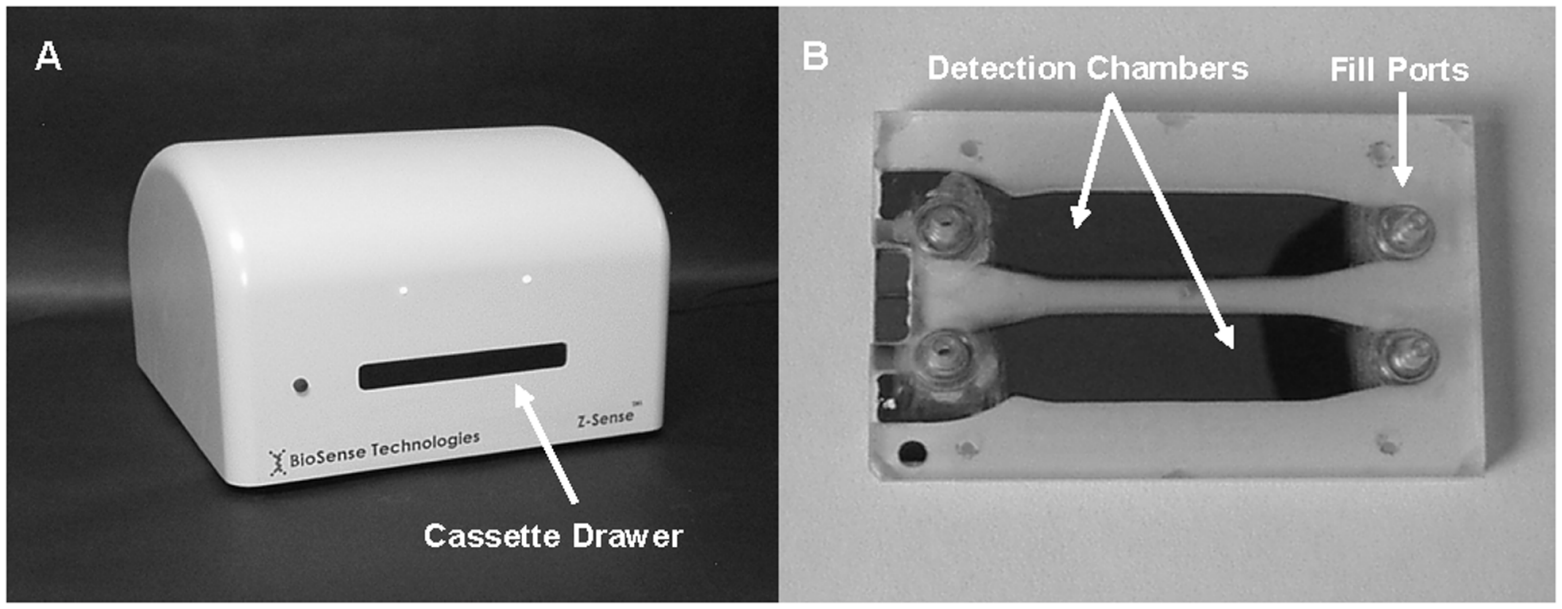
Photograph of the BioSense differential impedance sensing platform. The platform is comprised of an impedance analyzer (A) and a two-chamber test cassette (B, top view). The cassette is loaded into a drawer located on the face of the analyzer that extends and retracts and is designed to hold the cassette during the measurement.

All measurements of the dielectric permittivity were made using custom-built instrumentation. The measurement system consisted of a cassette holding the test samples and a table-top sized device into which the cassette was inserted for analysis (Fig. 5). All cassettes used in these experiments contained two mechanically and thermally matched 100 µl test chambers. Each test chamber was defined by a sub-millimeter electrode gap structure that encompassed a sample and measured its electrical properties. All electrodes were made from pure gold sputtered onto borosilicate glass substrates.

The measuring device consisted of thermally controlled platens that surrounded the cassette and maintained a constant temperature with high precision. The magnitude and angle of the impedance vector for each detection chamber was continuously recorded over time with instrumentation developed in-house and stored electronically in a computer.

For the planar geometry used in the cassette design, the dielectric permittivity is directly proportional to the measured capacitance (obtained from the reactive component of the recorded impedance vector). For all experiments, the dielectric permittivity was calculated according to the equation C = ε (A/d) where ε is the dielectric permittivity of the suspension, A is the surface area of the electrodes, and d is the separation distance between the electrodes.

The two detection chambers are located in close proximity so that both are subjected to identical thermal/mechanical perturbations. This provides a means for minimizing signal changes that are unrelated to the bacterial response. For example, changes in the dielectric properties due to interactions between organic compounds in the medium occurring in the first chamber which are also common in the second chamber are suppressed. Any difference in signal remaining after comparison is quantified by a parameter defined as the “Normalized Impedance Response” (NIR). The value of the NIR is calculated by dividing the signal values from one chamber by the other at each recorded time point.

### Preparation and enumeration of bacterial culture

Aliquots of the bacterial cell cultures were stored in liquid nitrogen. For each set of experiments, a new vial of frozen cells was thawed and bacteria were grown on appropriate agar plates to form colonies. An isolated colony was inoculated into 4 ml of broth and incubated at 37°C with shaking (200 rpm) for 16 hrs. This overnight culture was then diluted (1∶10) with fresh medium and incubated for an additional 1.5 hrs allowing cells to enter the exponential stage of growth. The respective cultures were diluted with fresh broth targeting a final concentration of 1×10^6^ CFU/ml. The resulting suspension was used for parallel measurements of the impedance response (see below) and for cell enumeration. For cell counting, the suspension was divided into two equal parts, antibiotics were added to one of the parts, and both were incubated at 37°C for 60 min. Colony counts were performed using ten-fold serial dilutions by dropping 25 µl of suspension in quadruplicate onto an appropriate agar plate. In parallel, acridine orange stained cells were counted and checked for viability using fluorescent microscopy.

### Determination of the MIC

The microbroth dilution method was used to estimate the bacteria susceptibility in accordance with Clinical and Laboratory Standards Institute (CLSI) document M7-A7. The assay media used were LB broth for *E. coli* and TSB for *S. aureus*.

### Differential impedance measurements

The protocol used for measurements of the dielectric properties of the antibiotic-treated model systems was as follows: One chamber of the cassette was manually filled with a bacterial suspension containing antibiotic or no antibiotic, while the other chamber, acting as a reference, was filled with sterile medium only. The filled cassette was then inserted into the analyzer set at 37°C, the electrical signals from each chamber were continuously recorded, and the NIR was calculated. All experiments were repeated a minimum of three times using freshly prepared starting aliquots.

For NIR measurements of the heat-shocked cells, heated bacteria were spiked into fresh medium and immediately transferred into one test chamber of the cassette; the adjacent control chamber was filled with the same medium spiked with PBS. For experiments with Triton X-100 and H_2_O_2_, one test chamber was filled with the reagent-treated bacterial suspension while the reference chamber was filled with the medium supplemented with the same amount of Triton X-100 or peroxide.

Importantly, experiments with the antibiotic-resistant strains differed slightly from the other experiments. In these experiments one test chamber was filled with antibiotic-treated bacterial suspension as before. However, the reference chamber was filled with the same bacterial suspension as the test chamber but without adding antibiotic.

### Statistical analyses

Student's *t*-test was used to compare the measurements. P-values of less than 0.05 were considered to indicate significant differences.

## Supporting Information

Table S1 Mean NIR values for Figures 1-4.
(0.09 MB PDF)
