# Supplementary material for: Rapid Microbiological Testing: Monitoring the Development of Bacterial Stress
Source: PLoS One. 2010 Oct 14;5(10):e13374. doi: 10.1371/journal.pone.0013374 (PMC2954791; doi:10.1371/journal.pone.0013374)
Supplement: Table S1 — Mean NIR values for Figures 1- 4. (0.09 MB PDF) [file pone.0013374.s001.pdf]

SUPPLEMENTARY DATA (MEAN NIR VALUES FOR FIG 2-5)

FIG 2A: Antibiotic Treated *E. coli* (ATCC 700926)

Time (Min)	CTRL	EC-Growth	2.5 μg/ml GEN	5 μg/ml GEN	12.5 μg/ml GEN
0	1.00000 ± 0.00000	1.00000 ± 0.00000	1.00000 ± 0.00000	1.00000 ± 0.00000	1.00000 ± 0.00000
10	0.99996 ± 0.00017	1.00147 ± 0.00062	0.99791 ± 0.00186	0.99649 ± 0.00058	0.99383 ± 0.00208
20	0.99987 ± 0.00026	1.00287 ± 0.00118	0.99709 ± 0.00183	0.99473 ± 0.00030	0.99100 ± 0.00201
30	0.99988 ± 0.00025	1.00417 ± 0.00177	0.99631 ± 0.00160	0.99333 ± 0.00018	0.98945 ± 0.00208
40	0.99996 ± 0.00020	1.00519 ± 0.00160	0.99564 ± 0.00162	0.99236 ± 0.00024	0.98887 ± 0.00214
50	0.99997 ± 0.00014	1.00587 ± 0.00185	0.99515 ± 0.00154	0.99174 ± 0.00026	0.98810 ± 0.00220
60	1.00000 ± 0.00007	1.00745 ± 0.00060	0.99443 ± 0.00157	0.99108 ± 0.00029	0.98808 ± 0.00163

FIG 2B: Antibiotic Treated *S. aureus* (ATCC 29213)

Time (Min)	CTRL	SA-Growth	4μg/ml VAN	20μg/ml VAN
0	1.00000 ± 0.00000	1.00000 ± 0.00000	1.00000 ± 0.00000	1.00000 ± 0.00000
10	0.99995 ± 0.00008	1.00056 ± 0.00023	0.99770 ± 0.00122	0.99630 ± 0.00119
20	0.99983 ± 0.00049	1.00089 ± 0.00031	0.99665 ± 0.00176	0.99503 ± 0.00097
30	0.99982 ± 0.00048	1.00124 ± 0.00051	0.99591 ± 0.00176	0.99379 ± 0.00109
40	0.99989 ± 0.00047	1.00160 ± 0.00072	0.99545 ± 0.00193	0.99310 ± 0.00095
50	1.00002 ± 0.00069	1.00197 ± 0.00095	0.99486 ± 0.00191	0.99264 ± 0.00078
60	1.00007 ± 0.00079	1.00233 ± 0.00118	0.99448 ± 0.00280	0.99255 ± 0.00047

FIG 3A: Chemically Stressed *E. coli* (ATCC 700926)

Time (Min)	CTRL	TX-100	H_2O_2
0	1.00000 ± 0.00000	1.00000 ± 0.00000	1.00000 ± 0.00000
10	1.00003 ± 0.00028	0.99411 ± 0.00241	0.99117 ± 0.00183
20	1.00000 ± 0.00027	0.98608 ± 0.00367	0.98105 ± 0.00085
30	1.00002 ± 0.00021	0.98168 ± 0.00398	0.97307 ± 0.00224

FIG 3B: Chemically Stressed *S. aureus* (ATCC 29213)

Time (Min)	CTRL	TX-100	H_2O_2
0	1.00000 ± 0.00000	1.00000 ± 0.00000	1.00000 ± 0.00000
10	0.99999 ± 0.00006	0.99374 ± 0.00105	0.99092 ± 0.00219
20	0.99997 ± 0.00015	0.98842 ± 0.00267	0.98306 ± 0.00315
30	0.99993 ± 0.00027	0.98458 ± 0.00477	0.97654 ± 0.00437

FIG 4A: Heat Shocked *E. coli* (ATCC 700926)

Time (Min)	CTRL	45°C
0	1.00000 ± 0.00000	1.00000 ± 0.00000
10	0.99997 ± 0.00002	0.99015 ± 0.00259
20	0.99998 ± 0.00017	0.98255 ± 0.00466
30	1.00000 ± 0.00033	0.97634 ± 0.00635

FIG 4B: Heat Shocked *S. aureus* (ATCC 29213)

Time (Min)	CTRL	48°C
0	1.00000 ± 0.00000	1.00000 ± 0.00000
10	0.99998 ± 0.00013	0.99339 ± 0.00055
20	0.99997 ± 0.00024	0.99037 ± 0.00006
30	0.99999 ± 0.00026	0.98852 ± 0.00052

FIG 5A: CIP-Resistant *E. coli* (clinical isolate M61965)

Time (Min)	CTRL	45 ug/ml CIP	15ug/ml GEN
0	1.00000 ± 0.00000	1.00000 ± 0.00000	1.00000 ± 0.00000
10	0.99995 ± 0.00012	1.00011 ± 0.00012	0.99292 ± 0.00095
20	1.00003 ± 0.00050	1.00005 ± 0.00027	0.98932 ± 0.00187
30	1.00036 ± 0.00057	0.99989 ± 0.00057	0.98806 ± 0.00149

FIG 5B: METH Resistant *S. aureus* (ATCC BAA-44)

Time (Min)	CTRL	METH	VAN
0	1.00000 ± 0.00000	1.00000 ± 0.00000	1.00000 ± 0.00000
10	1.00015 ± 0.00027	1.00033 ± 0.00088	0.99655 ± 0.00137
20	1.00007 ± 0.00028	1.00038 ± 0.00132	0.99057 ± 0.00210
30	1.00013 ± 0.00043	1.00041 ± 0.00143	0.98797 ± 0.00194
